# Cardiopulmonary exercise testing is safe in hypertrophic cardiomyopathy – Incidence and outcomes of sustained ventricular arrhythmias in a large referral cohort

**DOI:** 10.1016/j.ahjo.2026.100771

**Published:** 2026-03-25

**Authors:** Ibolya Csecs, Keti Mamillo, Jeffrey B. Geske, Andrés Garzona-Navas, Kyle W. Klarich, Steve R. Ommen, Thomas G. Allison

**Affiliations:** a Section of Cardiovascular Medicine, Department of Internal Medicine, Yale School of Medicine, New Haven, CT, USA; b Department of Anesthesiology and Perioperative Medicine, Mayo Clinic, Rochester, MN, USA; c Department of Cardiovascular Medicine, Mayo Clinic, Rochester, MN, USA; d Department of Pediatric and Adolescent Medicine, Division of Pediatric Cardiology, Mayo Clinic, Rochester, MN, USA

Cardiopulmonary exercise testing (CPET) is part of the standard clinical evaluation in hypertrophic cardiomyopathy (HCM) [1], [2]. A major focus of clinical management in HCM is risk stratification for life-threatening ventricular arrhythmias [1], [3]. Sustained and non-sustained ventricular tachycardias (VT) evidenced during Holter monitoring are associated with a higher risk of mortality [4]. In HCM, including in high-risk patients with prior cardiac arrest, VT, or syncope, there is evidence that exercise stress testing is safe and provides information on the severity and mechanism of functional limitation [5]. Particularly when combined with simultaneous analysis of respiratory gases such as CPET, exercise testing is strongly prognostic of future adverse cardiovascular events [1], [2]. CPET provides integrative physiological information that helps characterize the mechanisms underlying exercise intolerance and contributes to individualized risk stratification and therapeutic decision-making. However, limited data is available on the incidence of adverse events during CPET in this high-risk population.

We aim to share evidence from a large referral HCM center's CPET laboratory on the safety of exercise testing based on a cohort including 7637 CPET evaluations.

An institutional Integrated Stress Center database was reviewed for CPET testing performed on HCM patients between January 1994 and April 2017. All CPETs were conducted on the treadmill to limit symptoms using either the Mayo accelerated Naughton protocol or the standard Naughton protocol [6]. CPET variables were measured using MGD Diagnostics equipment as previously described [7]. Patients were referred for clinical CPET by a physician and medications were continued for the duration of test. Electronic medical records were reviewed for clinical information and follow-up data.

A total of 7637 CPETs were performed on HCM patients during the study period. The average age was 51 ± 16 years, 2900 (38%) were female. One-third of the cohort had hypertension, 3437 (45%) obesity, and 450 (5.9%) diabetes mellitus. Mean left ventricular ejection fraction assessed with echocardiography was 68% ± 11%. A total of 649 (8.5%) of the cohort had documented coronary artery disease while 1069 (14%) of patients underwent prior septal myectomy or alcohol ablation. Regarding arrhythmia history, 1069 (14%) had atrial fibrillation/flutter, and 535 (7%) left bundle branch block.

CPET termination was due to symptoms in the vast majority of studies 7179 tests (94%): 4200 (55%) secondaries to dyspnea, 993 (13%) to chest pain, and 320 (4.2%) because of dizziness. Peak heart rate (HR) was 135 ± 26 beats/min, peak respiratory exchange ratio 1.16 ± 0.11, and peak VO2 21.4 ± 7.4 mL/kg/min (68 ± 22% of predicted). Hypotension, defined as systolic blood pressure (SBP) <100 mmHg, was present in 1146 (15%) of cases at rest and 519 (6.8%) at peak exercise. In addition, 955 (12.5%) of patients had inappropriate blood pressure response as SBP failed to increase >10 mmHg.

Patients were considered to be at increased risk for VT during CPET if they had a history of any of the following: cardiogenic syncope, documented VT, history of aborted SCD, or prior ICD implantation (Fig. 1A). High risk for VT comprised 2642 (34.6%) of the tests, relatively high proportion, 764 (10%) of subjects with CPETs had a history of VT and 122 (1.6%) had history of aborted sudden cardiac death. The observed higher-risk population may be due to a combination of factors, including the complex pathophysiology of the referral population at our institution and possible clinical bias towards more testing of sicker patients. During CPET, 145 patients (1.9%) developed non-sustained VT (Fig. 1A). Compared with those without nsVT, patients with nsVT were more frequently male (81.4% vs. 61.6%, *p* = 0.001), with no differences in age, BMI, or comorbidities. Symptom occurrence was similar between groups during CPET; however, the nsVT group achieved higher peak HR and higher predicted peak VO₂. These findings suggest that patients who developed nsVT during CPET likely reached a higher exercise intensity, as reflected by higher peak heart rate and predicted peak VO₂, which may have contributed to the occurrence of nsVT. In addition, sustained ventricular tachycardia (SusVT) occurred on three tests (0.039%) (Fig. 1A). Among those three cases, one patient had a self-terminating fascicular VT that did not interrupt the test. However, the other two patients (Fig. 1B) collapsed and required resuscitation. The events occurred during sub-maximal exercise at 6 METs in each case. Both patients were young females with non-obstructive HCM on beta-blockade and had a strong family history of SCD. Patient 1 received a primary prevention ICD while Patient 2 had an out of hospital cardiac arrest at the age of 18 and subsequently received an ICD and was taking oral amiodarone.

**Fig. 1:**
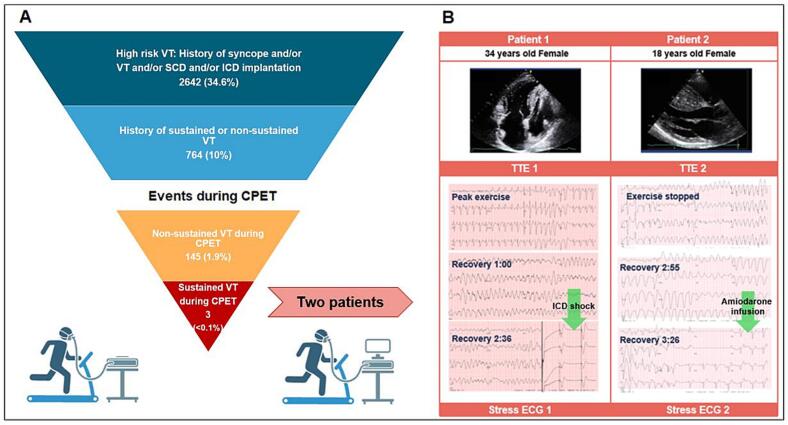
Adverse events incidence during cardiopulmonary exercise testing (CPET) in a large referral cohort of hypertrophic cardiomyopathy (HCM) patients. While as high as approximately one-third of the cohort had a higher risk to develop ventricular tachycardia (VT) (Panel A) defined as prior episode of VT or aborted sudden cardiac death or ICD, only a very small portion 145 (1.9%) developed VT during exercise stress testing and two among those patients collapsed and required resuscitation (Panel B). In Patient 1, sinus rhythm was restored after an ICD shock while in patient 2 the ICD failed to fire given the VT threshold was not met. Finally, prompt initiation of intravenous amiodarone resulted chemical cardioversion. CPET = cardiopulmonary exercise test; ICD = implantable cardioverter – defibrillator; SCD = sudden cardiac death; VT = ventricular tachycardia.

Patient 1 collapsed, and CPR had been initiated, but the VT was running under the ICD threshold rate. The team prepared to defibrillate externally when her VT threshold was ultimately reached, and a single shock terminated the arrhythmia after 2 min and 45 s. For Patient 2, the VT threshold was never reached therefore the ICD never fired. Intravenous amiodarone infusion was promptly started, and sinus rhythm was restored 3 min and 26 s after VT onset. The delay/failure in both cases was due to a low defibrillation threshold just below the rate of VT. These provoked and aborted VT episodes led to modification of ICD settings which could potentially prevent fatal outcomes in an out-of-hospital setting. To date, both patients are alive, one has undergone cardiac transplantation, while the other patient has remained stable and her listing for transplantation has been deferred.

The hemodynamic stresses associated with daily life and physical activity cannot be avoided in patients with HCM. The cardiovascular evaluation and risk assessment within a controlled and medically supervised CPET environment may facilitate timely identification of arrhythmic vulnerability under carefully monitored conditions, where immediate intervention is readily available. Avoidance of exercise testing does not mitigate the underlying risk but may instead limit opportunities for structured risk assessment and optimization of management. In a large referral cohort of HCM patients, VT/VF, was a rare finding during CPET. These results endorse the safety and importance of guideline-directed exercise stress testing in HCM patients. Importantly, the controlled CPET environment allowed immediate recognition and management of malignant arrhythmias and provided clinically actionable information, including optimization of ICD programming. These findings suggest that the diagnostic and prognostic value of CPET appears to outweigh its procedural risk when performed in experienced centers under appropriate supervision.

## CRediT authorship contribution statement

**Ibolya Csecs:** Writing – review & editing, Writing – original draft, Supervision, Conceptualization. **Keti Mamillo:** Writing – review & editing, Writing – original draft, Conceptualization. **Jeffrey B. Geske:** Writing – review & editing, Supervision. **Andrés Garzona-Navas:** Writing – review & editing, Writing – original draft, Conceptualization. **Kyle W. Klarich:** Writing – review & editing, Supervision. **Steve R. Ommen:** Writing – review & editing, Supervision. **Thomas G. Allison:** Writing – review & editing, Supervision.

## Ethical statement

This study was conducted in accordance with the ethical standards of responsible research practice. The study was retrospective in nature and used fully anonymized data; therefore, informed consent was not required.

## Declaration of competing interest

The authors declare that they have no known competing financial interests or personal relationships that could have appeared to influence the work reported in this paper.
